# Aligning Addiction Policy With Physiological Safety: The "Renal Trap" of Opioid Maintenance Therapy in Vulnerable Patients

**DOI:** 10.34172/ijhpm.9804

**Published:** 2026-08-22

**Authors:** Mohammad Moshiri, Reza Salehi Manzari, Bita Dadpour, Hamid Reza Fathi

**Affiliations:** ^1^Medical Toxicology Research Center, Faculty of Medicine, Mashhad University of Medical Sciences, Mashhad, Iran.; ^2^Kidney Transplantation Complications Research Center, Mashhad University of Medical Sciences, Mashhad, Iran.; ^3^Social Determinants of Health Research Center, Mashhad University of Medical Sciences, Mashhad, Iran.

## Introduction: Cultural Acceptance and the Misconception of Safety

 Opioid maintenance therapy (OMT) for opioid use disorder is globally dominated by methadone and buprenorphine as first-line agents, supported by robust evidence and endorsed by the World Health Organization (WHO). However, morphine-based formulations—though not standard treatment—persist in specific contexts: Slow-release oral morphine serves as a third-line alternative in some countries for patients who fail or cannot tolerate conventional agents.^1^

 Iran bears a substantial burden of substance use disorders, with prevalence estimates ranging from 3.85% to 11.05% across population subgroups.^2^ Opioids—particularly opium—occupy the dominant position among substances of abuse in this setting, driven by geographical proximity to major opium-producing regions and deeply rooted cultural perceptions of opium as a “natural” and beneficial remedy.^3^ In response, opium tincture (OT)—a complex formulation containing morphine alongside other alkaloids such as papaverine and noscapine in an alcohol base—has emerged as a widely utilized maintenance medication, employed as an alternative to methadone and buprenorphine. A systematic review of OT-assisted treatment reported treatment outcomes broadly comparable to methadone; however, critical appraisal reveals that nearly 90% of included studies failed to report adverse effects, and safety assessments were confined exclusively to short-term follow-up.^4^ Robust evidence regarding long-term adverse effects in aging patients is effectively absent. Iran’s widespread use of morphine-based maintenance serves as a sentinel context, revealing long-term physiological risks in aging populations that may remain obscured where prescription volumes are lower.

 Interpreting such “absence of evidence” as evidence of safety presents a significant clinical oversight, with implications extending beyond Iran to any setting where morphine-based therapy is employed in vulnerable populations—including slow-release oral morphine-based maintenance in European contexts and palliative care globally. As populations age, renal senescence, altered blood-brain barrier permeability and polypharmacy confer distinct and under-recognized risks in elderly patients receiving chronic morphine or OT; we therefore propose replacing the prevailing “one-size-fits-all” approach with a mechanism-based framework for risk stratification.

## Scope and Definitions

 We propose a hypothesis rather than test one, and do not question OMT’s effectiveness or advocate restricting access.^1^ *Elderly* denotes age ≥65, though we propose ≥60 as an operational monitoring threshold, renal senescence being continuous and regional cohorts accruing long opioid exposure earlier; *vulnerable patients*, those in whom ageing, renal impairment, multimorbidity, or polypharmacy narrows the therapeutic margin; *renal vulnerability*, reduced renal reserve, measured (estimated glomerular filtration rate [eGFR] <60 mL/min/1.73 m^2^) or anticipated; *physiological safety*, alignment of prescribing with actual organ capacity rather than protocol adherence. The “Renal Trap” is a hypothesis-generating model, not an established syndrome: declining renal function raises exposure to renally cleared morphine metabolites, while the resulting harm is slow, silent and attributed to ageing rather than treatment, so it never triggers the prescribing review that would interrupt it. Evidence was identified in PubMed/MEDLINE, Scopus, and Google Scholar since 2015 and selected purposively; claims are labeled by evidence type.

## The Mechanistic “Perfect Storm” in the Elderly

 The prevailing perception of OT’s safety—and indeed of opium and its primary alkaloid, morphine—cannot be extrapolated from younger adults to the elderly. Aging creates a “perfect storm” for opioid toxicity through several converging mechanisms (Figure), each established in geriatric clinical pharmacology; what is not established is the magnitude of their combined effect during long-term maintenance dosing.

**Figure:**
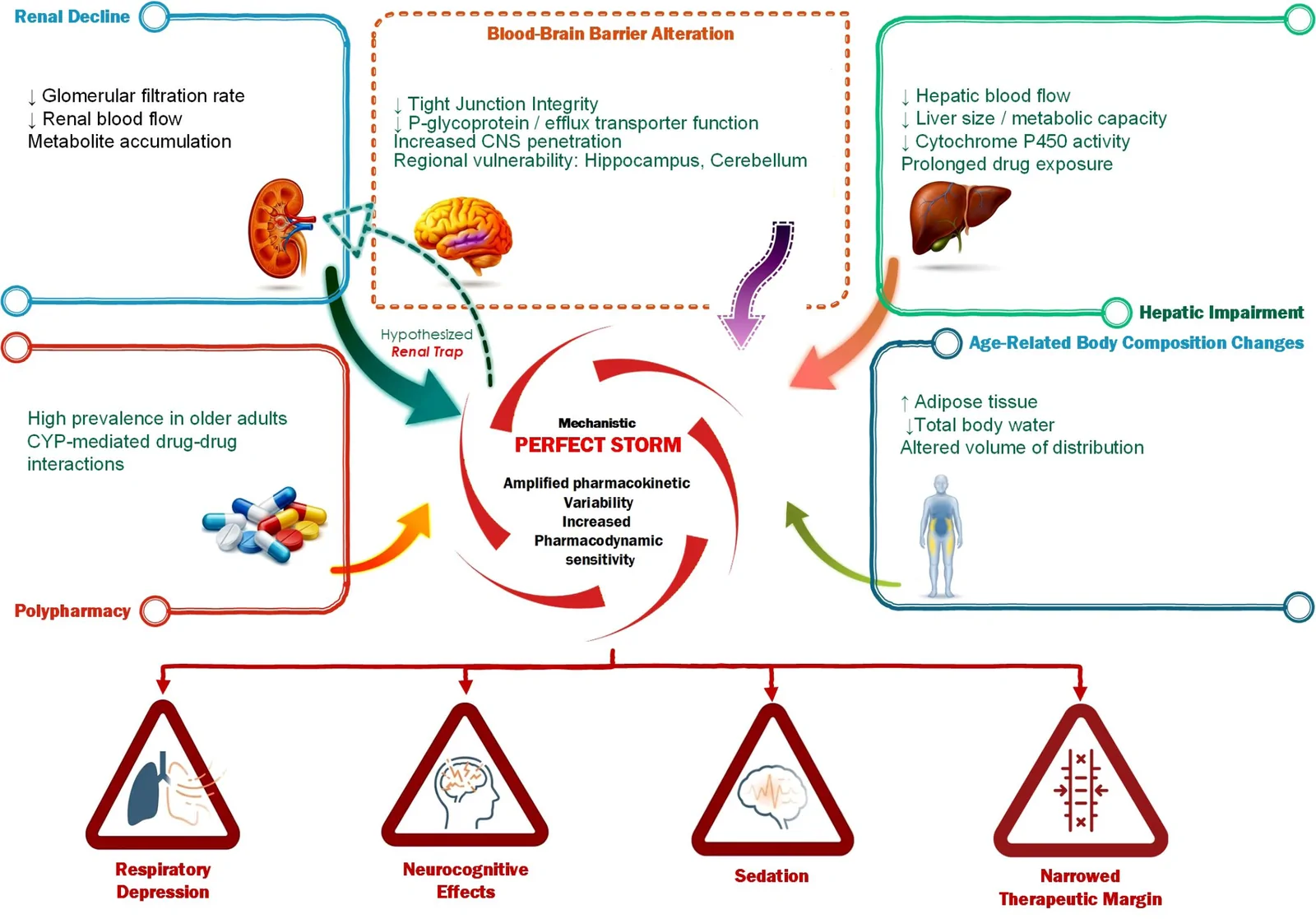
Schematic Depiction of Age-Related Factors Amplifying Opioid Pharmacokinetic Variability and Pharmacodynamic Sensitivity. Solid outlines denote evidence established in humans; dashed outlines denote hypothesized or non-human evidence. Arrows indicate proposed influence, not demonstrated causation. Abbreviations: CNS, central nervous system; CYP, cytochrome P450.

 Progressive renal decline, characterized by reduced glomerular filtration rate (0.5–1.0 mL/min/year) and renal blood flow, impairs the clearance of active morphine metabolites; morphine-6-glucuronide accumulates in patients with renal impairment, reaching cerebrospinal fluid concentrations at least 15-fold higher than in those with normal renal function (from analgesia rather than OMT populations; directional rather than quantitative for maintenance dosing).^5,6^ This is particularly problematic where diabetes and hypertension accelerate renal deterioration.^5^ Simultaneously, hepatic metabolism becomes compromised through decreased liver size, reduced hepatic blood flow and diminished cytochrome P450 (CYP) activity, prolonging drug exposure.^7^

 Beyond these pharmacokinetic alterations, aging results in structural degradation of the blood-brain barrier, including loss of tight junction proteins and endothelial dysfunction.^8^ The age-related decline in P-glycoprotein and other efflux transporters further compromises the brain’s protective mechanisms, allowing greater opioid penetration into the central nervous system (predominantly preclinical evidence, with sparse human confirmation).^9^ This increased central nervous system exposure heightens the risk of opioid-induced confusion, delirium and sedation in elderly patients.^5^

 Age-related changes in body composition—decreased total body water (10%–15%) and increased adipose tissue (20%–40%)—alter drug distribution in opioid-class-specific ways. Morphine is relatively hydrophilic: contraction of total body water reduces its volume of distribution and raises plasma concentrations for a given dose, whereas expanded adipose tissue prolongs elimination of lipophilic opioids such as methadone and buprenorphine.^7^ For morphine, therefore, the dominant determinant of exposure in the elderly is declining renal elimination of its glucuronide metabolites rather than fat redistribution.

 Polypharmacy, prevalent in up to 84.5% of elderly patients, adds CYP-mediated drug-drug interactions that increase additive sedation, respiratory depression and cognitive impairment.^10^ Current guidelines remain limited: integrated pharmacokinetic-pharmacodynamic models for combined organ impairment are underdeveloped, and clinical trials systematically exclude frail elderly individuals.^11^ One distinction is essential throughout: increased parent-drug or metabolite exposure arising from reduced renal clearance is a pharmacokinetic phenomenon, and is not in itself evidence of direct nephrotoxicity.

## The Nephrotoxic Cascade: Mechanisms of Opioid-Induced Kidney Injury

 Opioid-associated kidney injury is a recognized but heterogeneous concern. Much of the direct clinical evidence derives from acute intoxication and non-medical use—overdose with hypoxia and hypotension, rhabdomyolysis, volume depletion, injection-related infection, and adulterants—rather than from patients stable on prescribed maintenance.^12,13^ We cite it to establish that the kidney is an opioid-sensitive organ, not to attribute these injuries to OMT. No cohort has reported long-term renal outcomes among elderly OT recipients; that absence is the premise of our argument.

## Morphine: Mitochondrial Dysfunction and Programmed Cell Death

 Morphine induces kidney injury in experimental systems through mitochondrial dysfunction and consequent oxidative stress,^12^ damaging tubular cells via overlapping cell-death pathways, with inflammation propagating injury toward chronic kidney disease^14^ (mechanistic and preclinical evidence, at exposures not comparable to therapeutic maintenance dosing). If these mechanisms operate at maintenance-level exposures, patients with already diminished renal reserve have the narrowest margin of safety—a conditional that remains untested. The experimental picture is also not uniformly adverse: opioid-receptor signaling exerts both nephroprotective and nephrotoxic effects in models of renal ischemia-reperfusion injury.^15^

## Opium Tincture: The Alkaloid Cocktail Complexity

 OT introduces nephrotoxic complexity beyond pure morphine. Alcohol is unlikely to be responsible: a meta-analysis of cohort studies found a higher incidence of proteinuria only at intakes of approximately 36 g/d or above, with lower intakes neutral or inversely associated—substantially exceeding the ethanol content of therapeutic OT doses.^16^ Attention therefore falls on the alkaloid mixture. Papaverine exerts dose-dependent antiproliferative activity, oxidative stress induction and cell-cycle disruption in vitro (in tumor cell lines at 50–300 µM, not in renal cells and not in vivo; preclinical, and not extrapolable to OT plasma concentrations).^17^ Whether OT alkaloids interact additively or synergistically for renal outcomes remains unknown.

## Comparative Safety: Renal Pharmacokinetic Considerations

 Where renal vulnerability is established, opioid choice becomes clinically meaningful. Buprenorphine, a partial μ-opioid receptor agonist with a ceiling effect on respiratory depression, has minimal renal clearance of active moieties and requires no dose adjustment in chronic kidney disease or dialysis; it is the agent least likely to accumulate as renal function declines.^18^ Methadone, a full μ-agonist, is not removed by dialysis and accumulates little in renal impairment, but carries a dose- and electrolyte-dependent risk of QT prolongation requiring surveillance.^13,18^ Morphine and OT depend most on renal elimination of active metabolites. These are differences in predicted drug handling, not demonstrated differences in renal outcomes: no head-to-head study has compared long-term renal outcomes among these agents in elderly patients. Table summarizes the comparison on that understanding.

**Table:** Comparative Renal Considerations for Opioid Maintenance Medications: Proposed Mechanisms, Evidentiary Status and Clinical Trade-offs

**Agent**	**Proposed Mechanism of Renal Concern (Evidence Level)**	**Renal Pharmacokinetic Considerations**	**Principal Clinical Trade-offs**
Morphine	Mitochondrial dysfunction → oxidative stress → inflammatory injury (mechanistic/preclinical)	Active metabolites (M3G, M6G) renally cleared and accumulate as eGFR falls; review at eGFR <30 mL/min/1.73 m^2^	Metabolite-related sedation, delirium, respiratory depression; titration difficult where renal function fluctuates
OT	Morphine-related mechanisms plus uncharacterized effects of the alkaloid mixture (morphine, papaverine, noscapine; ethanol vehicle); papaverine cytotoxicity shown in vitro in tumour cell lines at supratherapeutic concentrations (preclinical)	As for morphine, with additional variability from mixture composition; long-term renal outcome data absent	Greatest uncertainty in elderly and CKD; supply continuity constrained in some settings
Buprenorphine	No established direct nephrotoxicity; ceiling effect limits hypoxia-mediated injury (mechanistic; no comparative renal outcome data)	Minimal renal clearance of active moiety; dose adjustment generally not required in CKD or dialysis	Induction may precipitate withdrawal on transfer from full agonists; inadequate symptom control in some patients; access, cost and authorisation barriers
Methadone	Low renal dependence; QT prolongation risk (clinical evidence, well-described)	Not removed by dialysis; limited accumulation in renal impairment	Requires ECG surveillance and attention to electrolytes and interacting drugs; long half-life complicates titration in frail patients

Abbreviations: OT, opium tincture; CKD, chronic kidney disease; ECG, electrocardiogram; eGFR, estimated glomerular filtration rate; M3G, morphine-3-glucuronide; M6G, morphine-6-glucuronide. *Note: *Entries reflect pharmacokinetic reasoning and indirect evidence. No head-to-head study has compared long-term renal outcomes among these agents in elderly patients receiving maintenance treatment; the table is not a ranking of demonstrated renal safety.

## Closing the Gap Between Experience and Evidence

 The critical question is not whether current protocols should be abandoned, but how clinical reasoning should adapt as renal reserve declines. Where renal function is preserved, existing approaches remain broadly defensible, though clinicians should remain attentive to early markers of cumulative toxicity rather than treating protocol adherence as a proxy for safety.^4^ As kidney function deteriorates into the moderate impairment range, the therapeutic margin narrows and routine evaluation of eGFR becomes a prerequisite for safe prescribing—not merely to document renal status but to recalibrate therapeutic strategy.^19^

 For patients with established moderate-to-severe renal impairment, metabolite accumulation, altered drug distribution, and reduced physiologic reserve meaningfully constrain what can be safely tolerated,^19^ and clinical reasoning must pivot from routine continuation toward individualized reassessment, with specialist consultation becoming standard rather than exceptional.^18^

 In advanced renal dysfunction, transition toward an agent with more predictable renal handling—most often buprenorphine—warrants active consideration, but not as a default. Transition from OT or methadone risks precipitated withdrawal, inadequate symptom control, treatment discontinuation, relapse and overdose on reduced tolerance, and these risks concentrate in exactly this population: older adults with multimorbidity, polypharmacy, frailty and cognitive impairment. Any switch should therefore follow individualized benefit–risk assessment and shared decision-making, use an induction strategy appropriate to the baseline opioid, and preserve supervision and psychosocial support.^1^ Renal impairment shifts the balance of considerations; it does not itself mandate a change of medication.

 This reframing requires structural support. Deprescribing principles—planned dose reduction or discontinuation where potential harms outweigh benefits^20^—apply here as scheduled reassessment rather than withdrawal of treatment. Our proposals differ in evidential status: renal function-guided opioid selection is established clinical pharmacology; baseline and periodic eGFR (proposed 6–12 monthly from age 60 years, or at any age with diabetes, hypertension or eGFR <60, ordered by the OMT prescriber), medication review and a referral pathway at eGFR <30 are pragmatic proposals rather than validated requirements; registry reporting, service-level audit of whether renal function is documented at all, and preferential reimbursement are priorities for evaluation, contingent on budget-impact, cost-effectiveness and service-capacity analysis. Audit should ask whether renal function is documented, not whether prescribing matched a threshold, which would penalize individualized judgment.

 Feasibility also has a supply dimension: OT availability in Iran has been inconsistent, and where it is scarce patients may be switched by supply failure rather than clinical decision—and supply of the alternatives has not always been secure either. Equity requires, in any health system, that an alternative be reliably available before an incumbent is discouraged.

## Limitations

 This is a narrative, conceptual article: the literature was selected purposively, and we present no original patient-level data. Direct evidence on long-term renal outcomes in elderly recipients of OT or other morphine-based maintenance is absent—the premise of our argument and also its principal weakness. Much supporting evidence derives from adjacent populations (chronic kidney disease, palliative care, overdose, non-medical use) or preclinical models, and its generalizability to supervised maintenance is uncertain; observational associations remain open to confounding by indication, comorbidity and concurrent substance use. The thresholds and intervals we propose are unvalidated and of unknown cost-effectiveness.

## Conclusion

 In aging populations, the absence of acute toxicity signals does not equate to long-term safety. Renal senescence, blood-brain barrier dysfunction and polypharmacy converge to transform standard maintenance protocols into a plausible source of silent, cumulative hazard—the “Renal Trap”—though the model rests on mechanistic inference rather than clinical proof.

 The path forward demands coordinated action across three domains. In *research*, prospective cohorts with linked laboratory data should quantify long-term nephrotoxicity and characterize metabolite exposure during maintenance. In *education*, training must distinguish absence of reported harm from demonstrated safety. In *policy*, protocols and guidelines should make renal function a required element of opioid maintenance prescribing, and any recommended alternative must be reliably available and reimbursed before an incumbent is discouraged.

 OMT remains effective and must remain accessible. But where the kidney can no longer clear what we prescribe, safety must be established physiologically rather than assumed from protocol—and for the aging patient, that is where “first, do no harm” now applies.

## Disclosure of artificial intelligence (AI) use

 Claude (Anthropic) was used for English language editing and improvement of readability and for assistance in organizing the point-by-point response to reviewers. The Gemini image model (google) was used solely to generate decorative graphical icons (organ and tablet illustrations) placed within Figure. The conceptual design, structure, panel content, evidence labelling, and all text of Figure were created by the authors using Microsoft Visio. No scientific content, argument, interpretation, analysis, or conclusion was generated by AI. All AI output was reviewed and edited by the authors, who take full responsibility for the content of the manuscript.

## Ethical issues

 This manuscript is a viewpoint based entirely on previously published literature. It did not involve human participants, patient-level data, biological samples, or animal experiments, and no new data were collected or analyzed. Formal approval by an ethics committee was therefore not required, and no ethical code applies.

## Conflicts of interest

 Authors declare that they have no conflicts of interest.

## References

[1] Yakovenko I, Mukaneza Y, Germé K (2024). Management of opioid use disorder: 2024 update to the national clinical practice guideline. CMAJ.

[2] Masoudkabir F, Shafiee A, Heidari A (2024). Epidemiology of substance and opium use among adult residents of Tehran; a comprehensive report from Tehran cohort study (TeCS). BMC Psychiatry.

[3] Moradinazar M, Najafi F, Jalilian F (2020). Prevalence of drug use, alcohol consumption, cigarette smoking and measure of socioeconomic-related inequalities of drug use among Iranian people: findings from a national survey. Subst Abuse Treat Prev Policy.

[4] Noroozi A, Kebriaeezadeh A, Mirrahimi B (2021). Opium tincture-assisted treatment for opioid use disorder: a systematic review. J Subst Abuse Treat.

[5] Dolati S, Tarighat F, Pashazadeh F, Shahsavarinia K, Gholipouri S, Soleimanpour H (2020). The role of opioids in pain management in elderly patients with chronic kidney disease: a review article. Anesth Pain Med.

[6] Gronich N (2024). Central nervous system medications: pharmacokinetic and pharmacodynamic considerations for older adults. Drugs Aging.

[7] Ngcobo NN (2025). Influence of ageing on the pharmacodynamics and pharmacokinetics of chronically administered medicines in geriatric patients: a review. Clin Pharmacokinet.

[8] Banks WA, Reed MJ, Logsdon AF, Rhea EM, Erickson MA (2021). Healthy aging and the blood-brain barrier. Nat Aging.

[9] Erdő F, Krajcsi P (2019). Age-related functional and expressional changes in efflux pathways at the blood-brain barrier. Front Aging Neurosci.

[10] Doan J, Zakrzewski-Jakubiak H, Roy J, Turgeon J, Tannenbaum C (2013). Prevalence and risk of potential cytochrome P450-mediated drug-drug interactions in older hospitalized patients with polypharmacy. Ann Pharmacother.

[11] Wu X, Valerie Sia JE, Hai M (2023). Physiologically based pharmacokinetic model for older adults and its application in geriatric drug research. Curr Drug Metab.

[12] Didik S, Golosova D, Xu B, Staruschenko A (2023). Opioids and the kidney: a compendium. Kidney360.

[13] Mallappallil M, Bajracharya S, Salifu M, Yap E (2021). Opioids and acute kidney injury. Semin Nephrol.

[14] Srivastava A, Tomar B, Sharma D, Rath SK (2023). Mitochondrial dysfunction and oxidative stress: role in chronic kidney disease. Life Sci.

[15] Gao S, He Q (2024). Opioids and the kidney: two sides of the same coin. Front Pharmacol.

[16] Yamamoto R, Li Q, Otsuki N (2023). A dose-dependent association between alcohol consumption and incidence of proteinuria and low glomerular filtration rate: a systematic review and meta-analysis of cohort studies. Nutrients.

[17] Gomes DA, Joubert AM, Visagie MH (2021). In vitro effects of papaverine on cell proliferation, reactive oxygen species, and cell cycle progression in cancer cells. Molecules.

[18] Roy PJ, Weltman M, Dember LM, Liebschutz J, Jhamb M (2020). Pain management in patients with chronic kidney disease and end-stage kidney disease. CurrOpin Nephrol Hypertens.

[19] Molnar AO, Bota SE, Naylor K (2022). Opioid prescribing practices in chronic kidney disease: a population-based cohort study. Nephrol Dial Transplant.

[20] Earl TR, Katapodis ND, Schneiderman SR, Shoemaker-Hunt SJ (2020). Using deprescribing practices and the Screening Tool of Older Persons’ Potentially Inappropriate Prescriptions criteria to reduce harm and preventable adverse drug events in older adults. J Patient Saf.

